# Efficacy of nano-carbonate apatite dentifrice in relief from dentine hypersensitivity following non-surgical periodontal therapy: a randomized controlled trial

**DOI:** 10.1186/s12903-020-01157-9

**Published:** 2020-06-12

**Authors:** Pei-Hui Ding, Anna Dai, Hua-Jiao Hu, Jia-Ping Huang, Jia-Mei Liu, Li-Li Chen

**Affiliations:** 1 Department of Periodontology, Affiliated Hospital of Stomatology, School of Medicine, Hangzhou, China; 2 Key Laboratory of Oral Biomedical Research of Zhejiang Province, Zhejiang University School of Stomatology, Hangzhou, China; 3 Department of Periodontology, the Second Affiliated Hospital of Zhejiang University School of Medicine, No. 88 Jiefang Road, Hangzhou, 310009 China

**Keywords:** Dentin hypersensitivity, Dentifrices, Randomized controlled trial, Periodontitis

## Abstract

**Background:**

Dentine hypersensitivity (DH) could occur or intensify after non-surgical periodontal therapy because of the exposure of dentine tubules, but currently no gold standard exists to treat DH. It has been demonstrated that nano-sized particles presented potential for dentine tubules blocking and remineralization. This randomized controlled trial aimed to investigate the efficacy of dentifrice containing nano-carbonate apatite (n-CAP) in reducing dentine hypersensitivity (DH) after non-surgical periodontal therapy.

**Methods:**

48 periodontitis patients with DH were included in this clinical trial. After non-surgical periodontal therapy, patients included were randomized to test and control group and the respective dentifrices were applied at chairside, after which they were instructed to brush teeth with the allocated dentifrices twice a day at home. Periodontal parameters were recorded at baseline and the last follow-up. DH was measured by air-blast test and recorded by visual analogue scale (VAS) and Schiff sensitivity scale at baseline, after polishing (0 week) and 2/4/6 weeks.

**Results:**

45 participants completed the follow-up. Periodontal parameters were improved and comparable between groups. Significant reduction in DH was observed in both groups at all time-points compared to baseline in terms of VAS and Schiff score. The test group achieved significantly greater relief from hypersensitivity compared with the control group after 4-week at-home use (for change of VAS, test group: 2.27 ± 2.47 versus control group: 1.68 ± 2.24, *p* = 0.036; for change of Schiff, test group: 0.94 ± 0.92 versus control group: 0.61 ± 0.83, *p* < 0.001). The 6-week results showed borderline significance between groups in terms of change of Schiff (*p* = 0.027) and no significance in terms of change of VAS (*p* = 0.256).

**Conclusions:**

Home-use of n-CAP based dentifrice had some benefit on alleviation of DH following non-surgical periodontal therapy after 4 weeks compared to the control product.

**Trial registration:**

Chinese Clinical Trials Registry (No. ChiCTR-IPR-17011678, http://www.chictr.org.cn/, registered 16 June, 2017).

## Background

Dentine hypersensitivity (DH) is characterized by an acute pain arising from exposed dentine in response to external stimuli, including thermal, evaporative, tactile, osmotic or chemical stimuli, which cannot be ascribed to other forms of dental defect or disease [1, 2]. The most widely accepted hypothesis to explain the mechanism of DH is Brannstrom’s hydrodynamic theory [3]. According to this theory, exposed tubules on dentine surface allow the rapid movement of dentinal fluid, indirectly stimulating the pulp nerve terminals and consequently causing sharp and shooting pain. Further histological study revealed that tubule diameters were significantly larger in hypersensitive area compared to non-sensitive surface which is usually covered by a smear layer [4].

DH can arise as a result of enamel loss caused by erosion, abrasion, attrition, as well as cementum loss typically subsequent to gingival recession [5]. Dental professionals may also contribute to cementum removal and tubule exposure by root surface instrumentations [6]. It is common that periodontitis patients complain about increased sensitivity following scaling and root planing [7]. The prevalence of DH in the published literature varies from 62.5 to 90% 1 day after non-surgical periodontal therapy [7]. In view of the high prevalence, a prophylaxis method to desensitize DH after root debridement procedure would be helpful for patients.

Over years numerous regiments have been recommended for the relief of DH, ranging from home-use desensitizing dentifrices and mouthwashes to in-office application products such as varnishes, dentine-bonding agents, composite resins, glass ionomer cements and laser [5]. Owing to low cost, easy use, home application and daily habit, desensitizing dentifrices could be considered as preferable agents in the routine management of DH. The Canadian Advisory Board on Dentine Hypersensitivity [2] suggested that home-care approach was the first choice to treat DH, such as desensitizing toothpaste. If the symptom was not alleviated, an in-office therapy would then be recommended.

Active ingredients like strontium [8] and potassium salts [9, 10] were widely used to act on the pulp nerve mechanoreceptors and block painful stimuli. However, DH might reoccur as the concentration of these ions decreased. The results in vitro demonstrated that several substances such as calcium chloride [11], fluoride [12], bioactive glass-ceramic [13] promoted the formation of a superficial pellicle over dentinal tubules, but this did not inevitably happen because the small particles could be dissolved or washed from the tubules in the in vivo environment after day-to-day activity [5]. Some studies have revealed that nano-sized particles would adhere to the dentine surface increasingly because of high surface energy, indicating their potential for occluding tubules [14, 15]. More importantly, the remineralization potential of nano-sized particles enabled them to maintain continuous capacity for exposed dentine surface repair [14, 16, 17]. A recent meta-analysis showed that nano-hydroxyapatite [Ca_10_(PO_4_)_6_(OH)_2_] provided greater DH relief when compared to placebo or negative control [18]. Carbonate apatite [CAP, Ca_10_(PO_4_·CO_3_)_6_(OH)_2_], whose structure was modified from hydroxyapatite, is chemically similar to the main inorganic component of dentine [19]. A new dentifrice containing 20% nano-sized CAP (n-CAP) has shown to occlude dentinal tubules of 77.4% more than that of the control group in vitro, which indicated a potential use in DH [15]. A recent clinical trial initially reported the desensitizing effect of this dentifrice in 2 weeks and the effect could be maintained for the later 2 weeks [20].

However, no published study has yet evaluated the effect of n-CAP in treating DH that occurred after scaling and root planing therapy. The present double-blind randomized controlled trial aimed to investigate the efficacy of the dentifrice containing 20% n-CAP in reducing DH following non-surgical periodontal therapy after immediate in-office application and during the 6-week home-use period.

## Methods

This randomized, controlled, parallel-group clinical trial was performed from June 2017 to July 2018 in the Department of Periodontology of the Second Affiliated Hospital of Zhejiang University School of Medicine, Hangzhou (SAHZU), China. This randomized trial followed the CONSORT 2010 Checklist protocol [21]. The study protocol was approved by the Human Research Ethics Committee of SAHZU (NO. 2017037) and registered at the Chinese Clinical Trials Registry (No. ChiCTR-IPR-17011678, http://www.chictr.org.cn/) before patient enrolment and conducted in full accordance with the Declaration of Helsinki.

### Inclusion and exclusion criteria

Each participant was fully informed about the research and signed the written consent prior to enrollment. The patient inclusion criteria were: (1) aged 18 to 60 years; (2) good general health without systemic disease; (3) no smoking history or quitting smoking for at least 2 years; (4) with a diagnosis of moderate or severe periodontitis [22, 23], who needed to be treated by non-surgical periodontal treatment (scaling and root planing) in one session. Briefly, moderate periodontitis was diagnosed as clinical attachment level (CAL) = 3 or 4 mm and probing depth (PD) ≥ 4 in ≥2 non-adjacent teeth, and severe periodontitis was diagnosed as CAL ≥ 5 and PD ≥ 5 in ≥2 non-adjacent teeth; (5) with at least three teeth in the buccal face existing dentine hypersensitivity [visual analogue scale (VAS) ≥ 2.0] after non-surgical periodontal therapy, which was evaluated by air-blast test. The patient exclusion criteria were: (1) patients with gross oral mucosal disease (oral lichen planus, oral ulcer, et al), reflux or bulimia, extremely advanced periodontitis who cannot endure one session non-surgical periodontal therapy; (2) patients receiving surgical or non-surgical periodontal therapy within 12 months; (3) patients using desensitizing agents in the past 6 months; (4) hypersensitive teeth with mobility greater than 1°; (5) hypersensitive teeth which were the second or third molars; (6) hypersensitive teeth with extensive and/or defective restorations, suspected caries, pulpitis or cracked enamel; (7) patients with chronic use of antihistamines, anticonvulsants, sedatives, antidepressants, tranquilizers or daily analgesics within 1 month; (8) pregnant or lactating females; (9) patients presenting allergies to the test product, or ever allergic to oral care consumer products; (9) patients who have participated in another desensitizing dentifrice study.

### Sample size estimation

The main outcome was the VAS difference across groups between the mean changes in air-blast test from baseline evaluation to the end of the follow-up. According to previous data [24, 25], the expected baseline mean VAS score was 5.5 ± 2.0. The hypothesized mean VAS score at the end of the follow-up was 2.0 ± 1.9 for the test dentifrice and 3.5 ± 1.8 for the control product, and the mean changes were 3.5 ± 1.8 and 2.0 ± 1.5, respectively. Using an unpaired *t*-test and assuming an α-error = 0.05, power = 80% (two-tailed comparison), a minimum of 21 participants per group were requested using a single allocation ratio (1:1) (G*Power version 3.1 for Mac, Franz Haul, University of Kiel, Germany). To compensate for the possibility of 15% dropouts, 48 patients per group were aimed to be recruited.

### Non-surgical periodontal therapy

The enrolled patients received full-mouth scaling and root planing in one session. The non-surgical periodontal therapy was performed using ultrasonic scaler (P5 Newtron, Acteon Satelec, France) combined with hand instruments (Gracey curettes, Hu-Friedy, Chicago, USA) after local anesthesia by 4% atticacaine and 1/100,000 adrenaline (Primacaine, Produits Dentaires Pierre Rolland, France). After the therapy, chairside irrigation with 0.12% Chlorhexidine (Koutai, South China Pharmaceutical, China) for 1 min. All patients were taught to brush teeth by modified bass technique using the same kind of soft toothbrush (Systema, Lion, Japan) provided, twice a day for 3 min during the 6-week trial.

All periodontal treatments were performed by the same experienced periodontist PHD, who was masked from the patient allocation during the whole study.

### Clinical evaluation

Baseline periodontal parameters were recorded before non-surgical periodontal therapy. Periodontal examination was performed for each tooth including gingival recession (GR), probing depth (PD), bleeding on probing (BOP) and clinical attachment level (CAL).

Baseline air-blast evaluation (post-scaling evaluation) for each tooth was taken 12–24 h after scaling and root planing. First, the tooth was isolated by the cotton roll from the adjacent teeth. Next, a blast of air from a standard dental unit syringe at 60 ± 5 psi at 18–22 °C was directed onto the exposed middle 1/3 buccal surface for 1 s at a distance of approximately10mm. Then, each patient reported the sensitivity he/she sensed using the Visual Analogue Scale (VAS) [26] and Schiff Cold Air Sensitivity scale [27]. VAS scale was scoring from 0 (no pain) to 10 (intense pain). Schiff scale was scored from 0 to 3: score “0” means no response, score “1” means response without request of discontinuation of stimulus, score “2” means response with request of discontinuation of stimulus, and score “3” means pain with request of discontinuation of stimuli.

According to the baseline evaluation, patients who presented at least three hypersensitive teeth with baseline VAS ≥ 2.0 were suitable to be included. 48 patients were sequentially enrolled by the dentist AD and randomly assigned to either test or control group.

Once enrolled, each patient would immediately receive one consecutive 5-s polishing of the assigned dentifrices by rubber cup at a moderate speed (about 1000 rpm) to all teeth by the same dentist JPH. The dentifrices were as follows: (1) test group: n-CAP dentifrice containing 20% n-CAP (Dentiguard Sensitive, Daewoong Co, Korea); (2) control group: calcium carbonate-based dentifrice free of n-CAP or other desensitizing ingredients (Honghua, Saky, China). Both of the dentifrices were without any form of fluoride. The two dentifrices had identical appearance, which were over-wrapped to hide their original packages and labeled with different numbers from 1 to 48. Neither investigators nor patients knew the codes. After application, each patient was subjected to post-polishing evaluation (0-week evaluation) of DH. Thereafter, patients would be called back to evaluate DH after 2, 4 and 6 weeks. At the 6-week follow-up, the same periodontal examination as baseline was repeated. Participants were instructed to use only the assigned products to brush teeth twice a day throughout the 6-week trial. They were also instructed not to eat acidic food before toothbrushing and 1 h before the DH evaluation. Participant adherence to dentine hypersensitivity treatment was evaluated by inquiry and examination of the remaining volume of dentifrice at each follow-up.

All periodontal and hypersensitivity outcomes were measured by the same experienced examiner HJH, who had been well-trained before patient enrolment. The same procedure as employed at baseline was used as the standardized method throughout the clinical trial. At each visit, the occurrence of potential adverse effects was assessed by investigators by both intraoral examination and patient inquiry.

### Randomization and allocation concealment

Simple randomization was adopted in this study. Random allocation list was generated using a computer program (Rand function, Excel 2016 for Mac, Microsoft, Redmond, VA, USA) by JML before patient recruitment. The assigned dentifrices were saved in opaque envelopes in advance. Other investigators only knew the number over the envelope but not the allocation sequence, and thus they were blind to the group allocation during the whole research period.

### Statistical analyses

The data of patients who adhered to the assigned intervention and completed the predefined process were included in the statistical analysis. Teeth with baseline VAS ≥ 2 and baseline Schiff score ≥ 1 were included. The normality of data was assessed by Shapiro-Wilk test. Data with skewness was converted through logarithmic conversion before analysis. Intragroup and intergroup comparisons were analyzed by paired *t* test and independent *t* test, respectively. The results of VAS and Schiff score were analyzed using the mixed linear model adjusted to age, gender, tooth type as well as the number of teeth per participant contributed to the air-blast test. The corresponding baseline results of VAS or Schiff were covariates. All statistical analyses were performed using SPSS (version 24.0 for Mac, Chicago, IL, USA). A two-tailed *p* value < 0.05 was considered significant. Primary outcomes were change of VAS and change of Schiff at all evaluation stages.

## Results

**Fig. 1:**
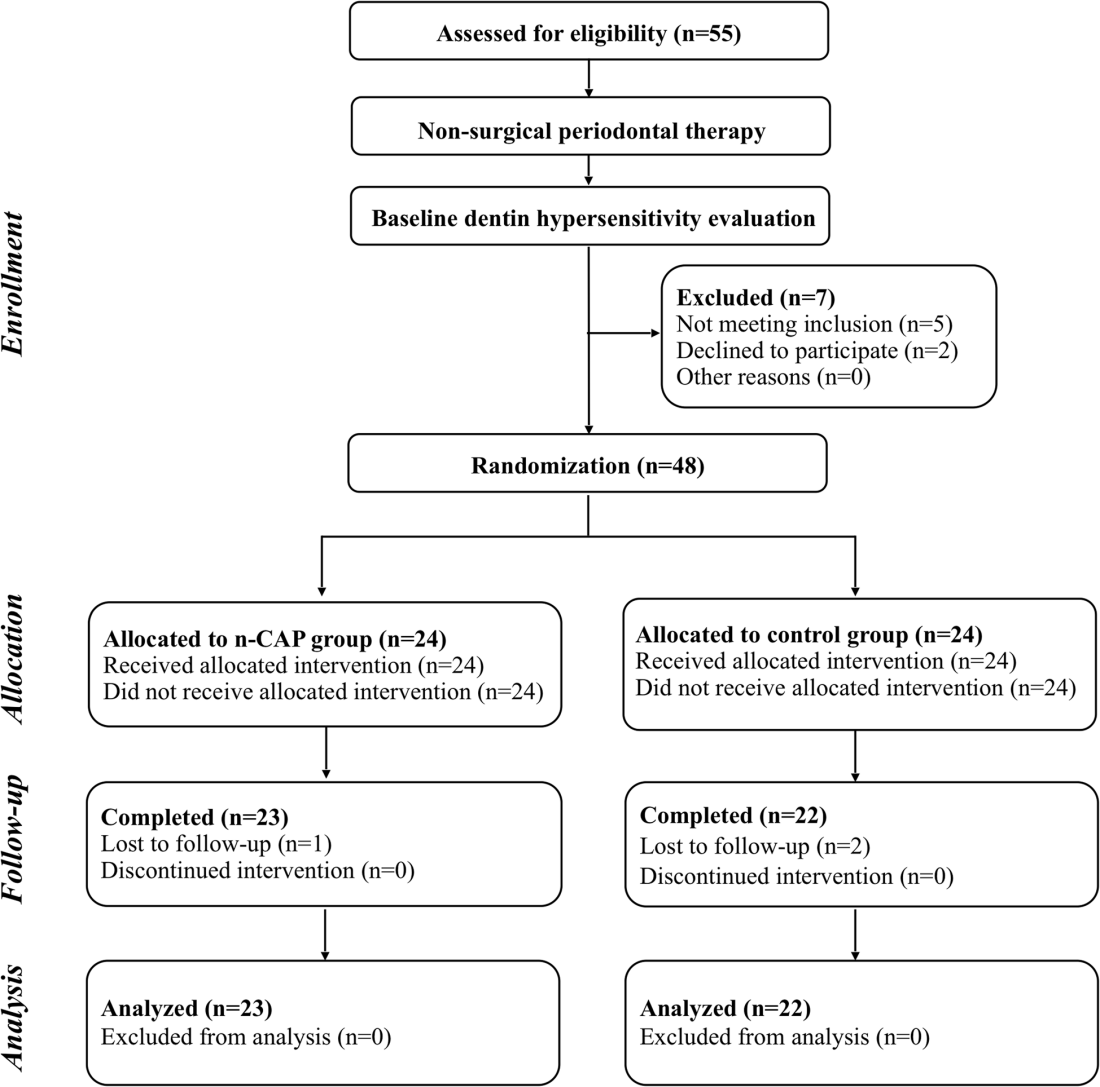
CONSORT flowchart of patients

Forty-eight patients (24 patients in each group) were initially included in the study and 45 participants completed the 6-week follow-up finally (Fig. 1). A total of 199 and 188 teeth were evaluated in the n-CAP and control group, respectively. Among the three patients who were lost to follow-up, two were unwilling to attend the follow-up for working hour limitation and the other one was unable to return to the hospital due to a car accident. The patient age (34.00 ± 7.63 versus 38.91 ± 7.96 years old for test and control), gender (ratio of male: 11/23 versus 9/22 for test and control) and number of hypersensitivity teeth for each participant were presented in Table S1.

The baseline periodontal parameters (CAL, PD, GR, BOP at patient level and cal, pd., gr at tooth level) were statistically comparable between the two groups (Table 1). The 6-week periodontal measurements (CAL, PD, BOP, cal, pd) were significantly reduced for both groups compared with the baseline measurements (*p* < 0.001). The outcomes of gingival recession were significantly increased for both groups (*p* < 0.001) and no statistical difference was shown between the groups (*p* > 0.05).

**Table 1:** Periodontal parameters in patient level (PD, GR, CAL, BOP) and hypersensitive tooth level (pd, gr, cal) by evaluation stage and group (mean ± standard deviation)

periodontal parameters								
**evaluation stage**	**group**	**patient level (*n*** **= 45)**				**hypersensitive tooth level (*n*** **= 387)**		
		**PD (mm)**	**GR (mm)**	**CAL (mm)**	**BOP (%)**	**pd (mm)**	**gr (mm)**	**cal (mm)**
**baseline**	**test**	3.65 ± 1.16^#^	0.84 ± 1.00^#^	4.50 ± 1.52^#^	41.03 ± 20.86^#^	3.60 ± 1.23^#^	1.16 ± 0.99^#^	4.74 ± 1.45^#^
	**control**	3.73 ± 1.19	0.90 ± 1.02	4.63 ± 1.52	35.53 ± 13.17	3.44 ± 1.10	1.23 ± 0.95	4.75 ± 1.30
**6-weeks**	**test**	2.68 ± 0.71^#,***^	1.06 ± 1.05^#,***^	3.74 ± 1.36^#,***^	19.19 ± 8.91^#,***^	2.53 ± 0.69^#,***^	1.33 ± 0.95^#,***^	3.82 ± 1.21^#,***^
	**control**	2.74 ± 0.86^***^	1.14 ± 1.10^***^	3.90 ± 1.44^***^	20.73 ± 6.45^***^	2.52 ± 0.68^***^	1.47 ± 1.16^***^	4.01 ± 1.30^***^

*PD & pd* pocket depth, *GR & gr* gingival recession, *CAL & cal* clinical attachment loss, *BOP* bleeding on probing

^#^: not statistically significantly different from the control group by independent *t* test (*p* > 0.05)

^***^: statistically significantly different from baseline by paired *t* test (*p* < 0.001)

**Fig. 2:**
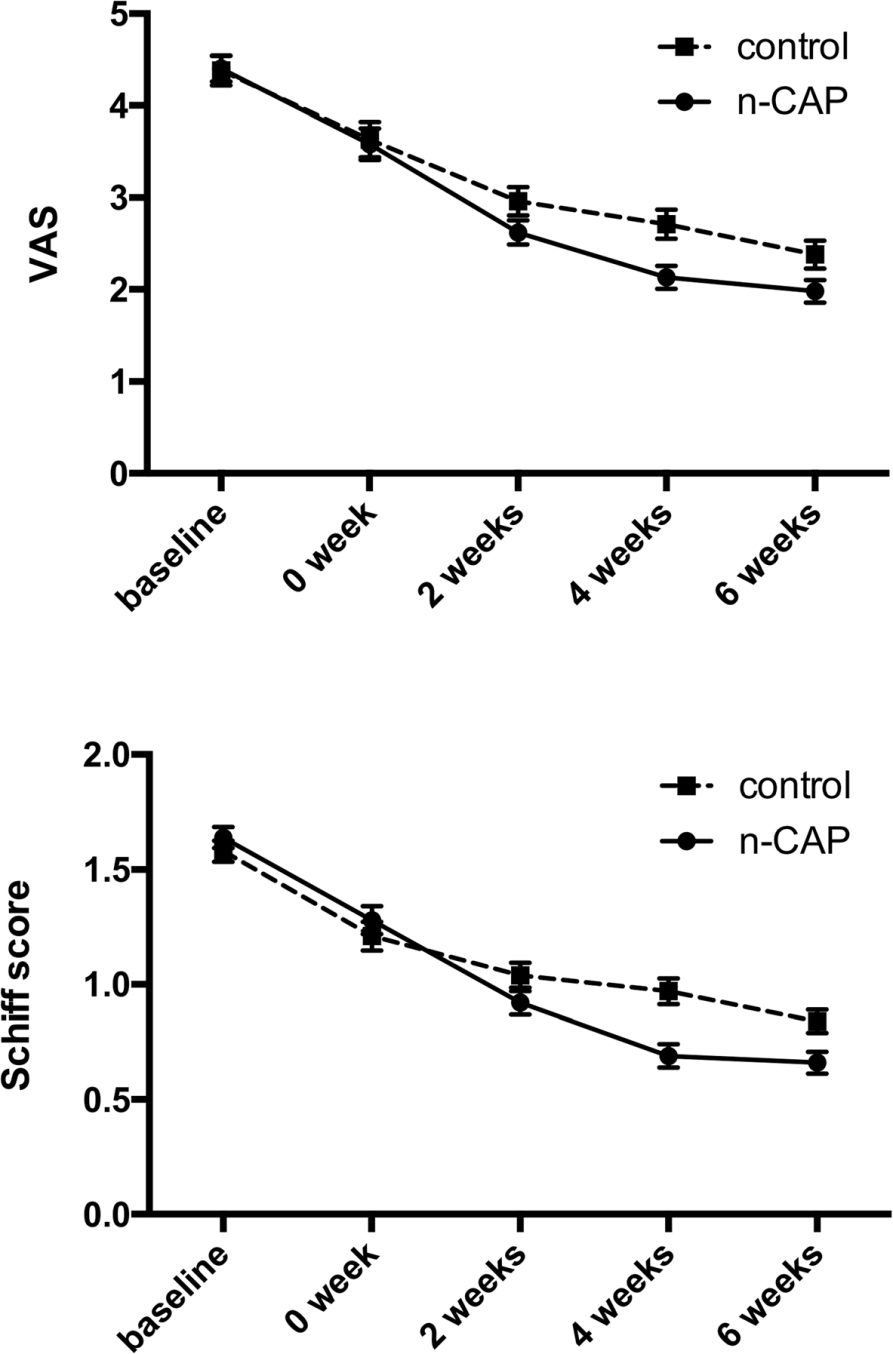
Hypersensitivity evaluation by air-blast test by treatment group and evaluation stage: **a** mean VAS (baseline ≥2); **b** mean Schiff score (baseline ≥1). Data shown are mean ± standard error

DH of each tooth was tested by air stimuli and recorded through VAS and Schiff scores. VAS and Schiff scores demonstrated no statistically significant difference at baseline between test and control groups (*p* > 0.05; Table 2, Table 3). For the 0-, 2-, 4- and 6-week evaluation, the reduction patterns of VAS and Schiff scores showed a similar trend and a significant desensitizing result in both groups (Fig. 2).

**Table 2:** Hypersensitivity evaluation by visual analogue scale (VAS) and change of VAS scores by evaluation stage and group (mean and standard deviation)

Evaluation stage	Mean (SD)		
	test (*n* = 199)	ctrl (*n* = 188)	*p*
**post-scaling (baseline)**	4.40 ± 1.96	4.38 ± 2.23	0.820
**post-polishing (0 week)**	3.58 ± 2.40 ^†^	3.63 ± 2.63 ^†^	0.800
**2 weeks**	2.62 ± 1.85 ^†^	2.96 ± 2.12 ^†^	0.197
**4 weeks**	2.13 ± 1.76 ^†^	2.71 ± 2.17 ^†^	0.005^**^
**6 weeks**	1.98 ± 1.72 ^†^	2.38 ± 2.10 ^†^	0.098
**change of VAS 0**	0.83 ± 1.95	0.76 ± 1.64	0.818
**change of VAS 2**	1.77 ± 2.11	1.43 ± 2.14	0.344
**change of VAS 4**	2.27 ± 2.47	1.68 ± 2.24	0.036^*^
**change of VAS 6**	2.42 ± 2.35	2.01 ± 2.27	0.256

^†^: statistically significantly different from baseline VAS by paired *t* test (*p* < 0.001)

^*^: statistically significantly different from control group by mixed linear model (*p* < 0.05);

^**^: statistically significantly different from control group by mixed linear model (*p* < 0.01)

**Table 3:** Hypersensitivity evaluation by Schiff score and change of Schiff scores by evaluation stage and group (mean and standard deviation)

Evaluation stage	Mean (SD)		
	test (n = 199)	ctrl (n = 188)	*p*
**post-scaling (baseline)**	1.64 ± 0.64	1.58 ± 0.63	0.457
**post-polishing (0 week)**	1.28 ± 0.85 ^†^	1.21 ± 0.85 ^†^	0.574
**2 weeks**	0.92 ± 0.71 ^†^	1.04 ± 0.75 ^†^	0.200
**4 weeks**	0.69 ± 0.71 ^†^	0.97 ± 0.77 ^†^	0.000^***^
**6 weeks**	0.66 ± 0.68 ^†^	0.84 ± 0.78 ^†^	0.047^*^
**change of Schiff 0**	0.36 ± 0.72	0.37 ± 0.64	0.973
**change of Schiff 2**	0.72 ± 0.81	0.54 ± 0.78	0.080
**change of Schiff 4**	0.94 ± 0.92	0.61 ± 0.83	0.000^***^
**change of Schiff 6**	0.97 ± 0.90	0.74 ± 0.80	0.027^*^

^†^: statistically significantly different from baseline Schiff score by paired *t* test (*p* < 0.001)

^*^: statistically significantly different from control group by mixed linear model (*p* < 0.05);

^***^: statistically significantly different from control group by mixed linear model (*p* < 0.001)

When comparing the VAS results between groups, test group showed significantly greater relief in DH than control group in the 4-week evaluation (*p* = 0.005). The hypersensitivity change of VAS for those sites was also statistically significant between groups at 4-week evaluation (*p* = 0.036). However, after 6 weeks, there was no significance between groups in terms of change of VAS (*p* = 0.256). For the results of Schiff score, test group showed more reduction in DH after 4 weeks (*p* < 0.001) and 6 weeks (*p* = 0.027) and the hypersensitivity change of Schiff score showed similar results.

No adverse effects on oral tissues observed or reported by participants throughout the 6-week follow-up.

## Discussion

The present clinical trial investigated the efficacy of n-CAP dentifrice in desensitizing hypersensitivity after non-surgical periodontal therapy compared with a control dentifrice in the continuous 6-week follow-up. Statistically significant decrease in DH was observed immediately after in-office application compared to baseline in both groups, while there is no difference between the test and control groups. The use of n-CAP could provide a significant reduction of DH after 4-week home-use. The 6-week comparison between groups only showed borderline significance.

Baseline and final (6 weeks) periodontal parameters (BOP, PD, GR, CAL, pd., gr, cal) and baseline air-blast hypersensitivity were measured. The results between test and control groups at baseline were statistically comparable. Based on the 6-week periodontal measurement, non-surgical periodontal therapy led to significant and similar improvement of periodontitis for both groups. The absence of significant difference in buccal gingival recession at baseline and 6 weeks between groups implied that the root surface exposure area was comparable between the two groups.

To explore the immediate desensitizing effect of the dentifrices, hypersensitivity was measured at post-polishing stage. The result indicated that the use of n-CAP did not benefit a lot to the relief of hypersensitivity immediately. Though the nano-particles are of high affinity [15], this did not enable them to adhere to the dentinal surface and seal the tubules in a few minutes, so immediate relief was not achieved as expected in this study. Douglas de Oliveira et al [28] reported that toothpaste containing calcium phosphate nanoparticles presented immediate relief effect. Notably, there was no significant difference between the test and control groups regarding evaporative and cold stimuli in the immediate assessment. Some in-office desensitizing agents have been reported to present instant relief from DH. A toothpaste with 15% of a calcium sodium phosphosilicate for a single professional application could provide a significant reduction of tactile sensitivity [29]. Another desensitizing paste containing 8% arginine and calcium carbonate led to instant relief from DH after a single in-office application [24].

On the other hand, after 4-week consecutive home-use of the n-CAP dentifrice, with progressive deposit to the surface and remineralization open dentinal tubules, a stable mineralized layer would generate on the surface, thus minimizing the degree of DH. In the 4-week evaluation, the degree of tooth sensitivity went significantly lower in the test group than that in the control group. This is in line with the results of a recent systematic review, which showed that dentifrices containing nano-hydroxyapatite had a significantly greater desensitizing effect than placebo or negative products in terms of evaporative and tactile after 4-week or 3-month follow-up [18]. Another dentifrice containing zinc-carbonate hydroxyapatite nanocrystals was also reported effective, which led to a significant reduction of the air-blast test score (mean percentage of reduction of 46% from baseline to 8-week evaluation) [30].

Interestingly, 6-week comparison between groups did not showed further significance than that of 4-week evaluation, raising the question whether 4-week results in the test group has decreased to a low DH level, leaving a minimal margin for further relief. As original data showed, there were as much as 93.1% patients in test group had Schiff score ≤ 1 after 4-week home-use. It was also implied in the tendency from 4-week to 6-week of Fig. 2 where a flatter line stood between these two timepoints. As a consequence, DH level went lower consistently in the control group while limited improvement occurred in the test group after 4 weeks.

It was noticed that the control group showed a similar tendency of decrease in DH during the follow-up period. It may also be attributed to the placebo effect which was common in the clinical studies of desensitizing pastes. Placebo products could reduce hypersensitivity by as much as 40% from baseline and therefore have an effect on the efficacy measurement of test dentifrice [31]. Another factor may be the Hawthorne effect. Participants tended to pay more attention to hypersensitivity and report positive outcomes in both groups. These two effects cannot be totally eliminated since the intention of the study can hardly be concealed from the participants. Our results also provided some evidence for the view that DH tend to self-heal over time after non-surgical periodontal therapy [32], possibly as a result of natural dentine tubule occlusion.

Air-blast test was used to test hypersensitivity in this study because it would cause more frequent pain than the tactile stimuli and involved a wider area of dentine, which indicated that the air-blast test is a sensitive and reliable method to detecting the degree of hypersensitivity [33]. The air-blast stimulus could better mimic the practical situation, since patients experienced sensitivity from cold water, food or air more frequently than other stimuli. In addition, many studies showed correlation between the results of air-blast test and tactile test [29, 34–36]. It is recommended that at least two independent stimuli should be applied [37], so secondary outcomes such as tactile, cold water test or subjective questionnaire could provide more supporting evidence in assessing DH degree. VAS and Schiff scores were used to exchange pain from subjective sense to objective scale. The results of these two parameters to evaluate DH were similar in the present study. Pepelassi et al. [24] also noticed that there was a strong positive correlation between VAS and Schiff scores after periodontal treatment, with the Pearson Correlation Coefficient up to 0.931 at 6-week measurement.

One possible limitation of the present clinical trial is the lack of a positive control group. It has been recommended to set both negative and positive control groups [37], but in fact, the gold standard treatment for DH has not yet been established [38]. Pastes containing arginine [39–41], strontium acetate [30, 42] potassium ion [43, 44] or potassium nitrate [45–47] have been widely used as positive control, which were expected to facilitate rapid and considerable relief of DH. The limited sample size is another shortcoming of the present study, which may cause potential bias. Moreover, the relatively low DH level at baseline left a limited extent for improvement. Hence, clinical trials enrolling a larger number of patients with higher inclusion criteria of baseline DH are encouraged to confirm the present findings and determine whether this product could be recommended to the general population.

## Conclusions

Within all the limitations, this randomized controlled trial showed that the application of n-CAP-based dentifrice after non-surgical periodontal therapy could had some benefit on the reduction of DH after 4-week at-home use compared to the control dentifrice.

## Supplementary information

**Additional file 1: Table S1.** Characteristics of included patients.

## Data Availability

The datasets used during the current study are available from the corresponding author on reasonable request.

## Abbreviations

BOP: Bleeding on probing
CAL: Clinical attachment level;
DH: Dentine hypersensitivity
GR: Gingival recession
n-CAP: Nano-carbonate apatite
PD: Probing depth
SD: Standard deviation
VAS: Visual analogue scale
