# Supplementary material for: Efficacy of nano-carbonate apatite dentifrice in relief from dentine hypersensitivity following non-surgical periodontal therapy: a randomized controlled trial
Source: BMC Oral Health. 2020 Jun 12;20:170. doi: 10.1186/s12903-020-01157-9 (PMC7291678; doi:10.1186/s12903-020-01157-9)
Supplement: Supplementary file 1 — Additional file 1: Table S1. Characteristics of included patients. [file 12903_2020_1157_MOESM1_ESM.docx]

Table S1. Characteristics of included patients

| **number** | **group** | **age range (year)** | **included sites** |
| --- | --- | --- | --- |
| 1 | n-CAP | 31-40 | 10 |
| 2 | n-CAP | 21-30 | 4 |
| 3 | control | 41-50 | 9 |
| 4 | n-CAP | 21-30 | 7 |
| 5 | n-CAP | 21-30 | 8 |
| 6 | n-CAP | 31-40 | 6 |
| 7 | control | 51-60 | loss of follow-up |
| 8 | control | 31-40 | 14 |
| 9 | control | 41-50 | 9 |
| 10 | n-CAP | 21-30 | loss of follow-up |
| 11 | control | 31-40 | 6 |
| 12 | control | 51-60 | 4 |
| 13 | control | 21-30 | 5 |
| 14 | control | 31-40 | 6 |
| 15 | n-CAP | 31-40 | 6 |
| 16 | n-CAP | 21-30 | 3 |
| 17 | control | 31-40 | 3 |
| 18 | control | 41-50 | 9 |
| 19 | control | 21-30 | 6 |
| 20 | control | 41-50 | 8 |
| 21 | n-CAP | 21-30 | 17 |
| 22 | n-CAP | 21-30 | 8 |
| 23 | n-CAP | 31-40 | 13 |
| 24 | n-CAP | 31-40 | 10 |
| 25 | control | 51-60 | 8 |
| 26 | n-CAP | 41-50 | 4 |
| 27 | n-CAP | 31-40 | 12 |
| 28 | control | 41-50 | 9 |
| 29 | control | 51-60 | loss of follow-up |
| 30 | n-CAP | 51-60 | 7 |
| 31 | control | 31-40 | 9 |
| 32 | control | 31-40 | 12 |
| 33 | n-CAP | 41-50 | 7 |
| 34 | n-CAP | 21-30 | 5 |
| 35 | control | 41-50 | 8 |
| 36 | control | 41-50 | 7 |
| 37 | n-CAP | 41-50 | 12 |
| 38 | control | 41-50 | 13 |
| 39 | n-CAP | 21-30 | 13 |
| 40 | n-CAP | 31-40 | 10 |
| 41 | control | 21-30 | 10 |
| 42 | control | 31-40 | 12 |
| 43 | control | 21-30 | 13 |
| 44 | control | 31-40 | 8 |
| 45 | n-CAP | 31-40 | 10 |
| 46 | n-CAP | 31-40 | 16 |
| 47 | n-CAP | 31-40 | 6 |
| 48 | n-CAP | 31-40 | 5 |
